# Use of bisphosphonates in prostate cancer: Current status

**DOI:** 10.4103/0970-1591.30267

**Published:** 2007

**Authors:** Rishi Nayyar, Narmada P. Gupta

**Affiliations:** Department of Urology, All India Institute of Medical Sciences, New Delhi - 110 029, India

**Keywords:** Bisphosphonates, prostate cancer, hormone resistant prostate cancer

## Abstract

Bisphosphonates are a relatively newer class of drugs which have been used for the prevention of skeletal related complications of age related osteoporosis or metastatic disease from carcinoma breast or multiple myeloma. Their role in the management of prostate cancer is still under evolution. We hereby review the ongoing and some published randomized trials to define the role of bisphosphonates in various stages of prostate cancer.

Bisphosphonates are a relatively newer class of drugs which have been used for the prevention of skeletal-related complications of age-related osteoporosis or metastatic disease from carcinoma breast or multiple myeloma. Their role in the management of prostate cancer is still under evolution. We hereby review the ongoing and some published randomized trials to define the role of bisphosphonates in various stages of prostate cancer.

Since no curative therapy is available for patients with advanced carcinoma of the prostate, sooner or later such patients develop metastatic disease, most commonly to the bones. Between 1984 and 1991, 30 to 40% of men with prostate cancer presented with advanced disease, while at present, only 5% of men have metastases at initial presentation.[1] Bone involvement is a significant cause of morbidity and disability in these patients. Bisphosphonates is a relatively newer class of drugs which have been utilized routinely for bone involvement in patients with metastatic breast cancer and multiple myeloma. For these tumors they have been shown to reduce the risk of fractures and the need for palliative radiotherapy.[2] Their role in prostate cancer is relatively less well established. Presently their role in the treatment of prostate cancer is being assessed in several ongoing trials. They also have a potential role in treating and preventing androgen deprivation-induced osteoporosis. More recently, they are being used in combination regimens as part of multi-drug chemotherapy.

## MECHANISM OF ACTION

Bisphosphonates (previously called diphosphonates) are stable pyrophosphate analogs that have several actions including likely inhibition of osteoblast proliferation and differentiation, decreasing osteoclast differentiation and induction of apoptosis through caspase activation.[3]

They decrease bone resorption, primarily through direct inhibition of osteoclast activity and proliferation. Most of the metastases in prostate cancer are osteosclerotic, but it has been shown that the abnormal osteoblastic bone formation within metastases is preceded by osteoclastic activation, which appears to be associated with bone pain. This provides the rationale for using bisphosphonates, which are powerful and selective inhibitors of osteoclastic bone resorption. They have also been shown to have anti-tumoral effects on prostate cancer cell lines.[4]

The various available bisphosphonates are given in Table 1. They have been classified as 1^st^, 2^nd^ or 3^rd^ generation agents, based on the type of side chain on the carbon atom, their relative antiresorptive potencies and when they were developed. With each new generation has come a 10- to 100-fold increase in antiresorptive potency.

**Table 1:** Various available bisphosphonates

Bisphosphonate	Route of administration
3^rd^ generation	
Risedronate	oral
Zoledronate	iv
Neridronate	im
2^nd^ generation	
Alendronate	oral
Pamidronate	iv
1^st^ generation	
Clodronate	oral
Etidronate	oral

Bisphosphonates are poorly absorbed from the gastrointestinal tract. Typically, absorption ranges from 0.7% to 3% of an oral dose and is significantly reduced in the presence of calcium, other divalent cations or food or beverages other than plain water. Bisphosphonates are not metabolized. After binding to bone surfaces and exerting their effects on osteoclasts, they are retained in the bone for months or years and are slowly released with the process of bone turnover. The portion of absorbed drug that is not bound to bone is excreted by the kidney unchanged [Table 1].

### Role in preventing skeletal complications of androgen deprivation therapy in advanced nonmetastatic prostate cancer

Although, normal men also have a gradual age-related loss of bone mineral density (BMD) of 7-12% per decade beginning at age 30, primary male osteoporosis is not common.[5] But patients of advanced carcinoma prostate are at a relatively high risk of skeletal morbidity including pathological fractures because of frequent bone metastasis and the use of androgen deprivation therapy, which can hasten loss of BMD. Androgen suppression reduces BMD by approximately 3-7% per year[6] and such men have BMD measurements about 6.5-17.3% lower than men who do not receive androgen deprivation therapy.[7] Higher overall fracture rates of ^˜^9% have been reported in men with prostate cancer who are treated with androgen deprivation therapy.[8] This rate is about three times the expected incidence of fractures in normal men of similar age (TOWNSEND). Another recent study has reported a 19.4% fracture rate in prostate cancer patients receiving androgen deprivation therapy compared with 12.6% rate in patients not receiving it[9] (*P* < 0.001). Increased occurrence of hip or spine fractures has been related to increased morbidity as well as mortality in several existing reports.[10–12] Even in men who have hormone naïve prostate cancer and are not on androgen deprivation therapy, higher than expected rates of osteoporosis and decreased BMD have been seen.[13] In them, this decreased BMD correlated significantly with advanced age, lower body mass index and high PSA.

Several treatments have been tried to reduce bone mineral loss in cancer prostate patients including daily calcium, vitamin D, weight-bearing exercise, estrogen therapy, calcitonin and bisphosphonates. Low-dose estrogen therapy and transdermal estradiol therapy have been shown to reduce bone mineral loss with effective testosterone suppression and without significant cardiovascular or thromboembolic toxicity.[14,15] Other drugs have not shown much benefit so far except bisphosphonates, which have been proved in several randomized controlled trials to improve bone mineral density in cancer prostate patients.

Many bisphosphonates including pamidronate, zoledronic acid and neridronate have been shown to significantly improve the bone mineral density in spine, femoral neck, trochanter and hip as compared to a placebo in men with advanced nonmetastatic prostate cancer treated with androgen deprivation therapy.[16–18] The BMD actually increased in patients treated with androgen receptor antogonist therapy and there was no loss of BMD in patients treated with maximal androgen blockade. On the other hand, in placebo groups there was an increase in the urinary deoxypyridoline (DPD) and bone alkaline phosphatase, both of which are markers of increased bone turnover. Also, there was a definite bone loss as measured by the DEXA scan. Although no patient in these trials suffered from clinical (symptomatic) fracture, there were some radiographically detected new or worsening vertebral fractures in both the groups, whether taking bisphosphonates or not. The limitations of these studies include an overall quite short follow-up, up to one year. Moreover, none of the studies has so far evaluated the site-specific fracture rates and it remains to be seen whether preventing BMD loss in these patients translates into reduced symptomatic or clinically significant fracture rate.[19]

### Role in preventing skeletal complications in hormone naïve metastatic prostate cancer

Clodronate was shown to improve, although insignificantly, the overall survival of men with hormone naïve metastatic prostate cancer in MRC PR05 trial.[20] Another large multicenter, international randomized trial (the MRC Systemic Therapy in Advancing or Metastatic Prostate cancer: Evaluation of drug efficacy, STAMPEDE trial) has been set up to assess the safety and efficacy of zoledronic acid in patients starting long-term androgen deprivation therapy. Its results should be available in the near future.

### Role in preventing skeletal complications in hormone resistant metastatic prostate cancer

Randomized trials done using clodronate 1500 mg vs. Placebo[21] and pamidronate 90 mg vs. placebo[22] in men with bone metastasis from HRPC, did not show any significant improvement in the pain or analgesic requirement or quality of life. But possible benefit was surely suggested for patients who had more severe pain as baseline at the start of the study. Moreover, these trials did not use third generation bisphosphonate (like zoledronic acid), which could have provided more pain relief. Another larger study done using zoledronic acid[23] and including 643 men with bony metastasis from hormone resistant prostate cancer (ZAPCSG, Zoledronic acid prostate cancer study group trial) showed a statistically significant reduction in skeletal-related events including pathological fractures, spinal cord compression, surgery or radiation therapy to bone and a change of systemic anticancer treatment (33.2% vs. 44.2%, *P* = 0.021). But even in this trial, the result did not translate into a significant benefit of quality of life though there was an insignificant trend towards improved overall survival for the zoledronate arm compared with placebo. In the absence of any statistically significant impact either on the quality of life or on overall survival, routine use of zoledronate cannot be recommended. The reduction in pain although said to be clinically significant for patients receiving zoledronate 4 mg, was said to be to the tune of only 0.47 units over a scale of 11 points, which is questionable.[24] Also bisphosphonates have there own side-effects including fatigue, anemia, myalgia, fever, edema, weight loss and renal toxicity. Sometimes these side-effects may be the reason for the discontinuation of the drug. These may also have a bearing on the relatively poor improvement in the overall quality of life, seen in the ZAPCSG trial. In the absence of a proven overall advantage if used in all patients with metastatic hormone-resistant prostate cancer, one needs to identify the subset of patients which may derive significant benefit from the use of bisphosphonates.[25] Such patients may include those who have more severe pain at the start of therapy or have poor BMD with associated risk of bone fracture or those who show striking benefit with the first few doses of the drug. The most beneficial dose and scheduling of these drugs are also still evolving with several ongoing trials. Although 8mg of zoledronic acid has been shown to provide better pain relief, it provided poorer efficacy in preventing skeletal-related events. The side-effects are even more with the higher dose of zoledronic acid.[23]

### Use in combination with chemotherapeutic agents as multi-drug chemotherapy

Various chemotherapy drugs are being tried in patients of hormone-resistant prostate cancer for their palliative benefit. However, the achievement of survival benefit has not been possible so far. Bisphosphonates, particularly zoledronic acid, has been shown to have an anti-tumoral synergistic interaction with taxanes both *in-vitro* and *in-vivo*.[26,27] Moreover, as discussed above, bisphosphonates may reduce skeletal-related events, besides reducing pain and analgesic requirement. They also have different toxicity profiles. Therefore the combined use of zoledronic acid with docetaxel and prednisone is being tried for its possible survival benefit and improvement of quality of life. One pilot report using this combination showed significant improvement in pain and reduction in analgesic requirement with a reduction in PSA by more than half in more than 50% of patients.[28] A similar trial is ongoing at our institute and the results are soon going to be analyzed.

### Adverse effects of bisphosphonate therapy

Bisphosphonates are generally well tolerated, but they too are associated with their attendant side-effects. Nausea and abdominal pain are common with oral bisphosphonates. Upper gastrointestinal disorders such as dysphagia, esophagitis, esophageal ulcer and gastric ulcer may occur. It is recommended that patients take bisphosphonates on an empty stomach with a full glass of water and remain in an upright position for at least 30 min after a dose. Acute phase reaction in the form of arthralgia, fever, transient leucopenia, bone pain, iritis etc. is commonly seen with intravenous administration but is usually self-limited. Nephrotic syndrome and jaw osteonecrosis have also been reported. Osteomalacia and hyperphosphatemia have been seen with etidronate. Other usual adverse effects include fatigue, anemia, myalgia, fever, edema, weight loss, skin rash and renal toxicity.

## SUMMARY

The current evidence suggests that bisphosphonates reduce the overall bone turnover and increase or maintain BMD in patients treated with androgen deprivation therapy. This stabilization of BMD may decrease the risk of fracture in these patients, which is otherwise associated with higher degree of morbidity and mortality. However, achievement of benefit in terms of improvement of quality of life and overall survival remains to be proven. Although no specific guidelines are available as yet, BMD measurement by DEXA scan should be strongly considered and bisphosphonate therapy should be instituted in cases with clinical evidence of decreased BMD or fracture or those who have severe pain at presentation.
